# Characteristics, Treatment and Outcomes of Stage I to III Colorectal Cancer in Patients Aged over 80 Years Old

**DOI:** 10.3390/cancers17020247

**Published:** 2025-01-14

**Authors:** Melissa R. Yeo, Ioannis A. Voutsadakis

**Affiliations:** 1 Northern Ontario School of Medicine, Sudbury, ON P3E 2C6, Canada; meyeo@nosm.ca; 2 Algoma District Cancer Program, Sault Area Hospital, Sault Ste. Marie, ON P6B 0A8, Canada; 3 Section of Internal Medicine, Division of Clinical Sciences, Northern Ontario School of Medicine, Sudbury, ON P3E 2C6, Canada

**Keywords:** colorectal cancer, octogenarians, geriatric patients, chemotherapy, radiation therapy, adjuvant

## Abstract

Background: Colorectal cancer primarily affects older adults and poses treatment challenges due to age-related comorbidities and frailty, which hinder surgical and chemotherapy options for many elderly patients. This study aims to analyze treatment and disease patterns in elderly colorectal cancer patients, aged over 80 years old, to inform personalized therapies that accommodate their unique clinical needs and improve their outcomes. Patients and Methods: The medical records of all patients aged 80 years old and above, and those aged 65 to 75 years old, who were diagnosed with colorectal cancer at a cancer center in Canada over a seven year period, were retrospectively reviewed. Results: No significant differences in the initial presentation, location, grade or stage at colorectal cancer diagnosis were observed between age groups. Patients aged 80 years old and above were less likely to receive neoadjuvant and adjuvant chemotherapy treatments for stage II disease (19.2% versus. 58.6%, *p* = 0.002; 7.9% versus. 40.0%, *p* = 0.002). There were also differences in the intensity of chemotherapy received and the frequency of dose reductions (76.0% vs. 10.0%, *p* = 0.0001), neoadjuvant and adjuvant radiation therapy (34.6% vs. 65.5%, *p* = 0.02) and surgical management (83.7% vs. 95.3%, *p* = 0.006). Despite these differences in treatments, recurrence rates were not statistically significant between the two groups. However, overall survival was reduced in the older age group. Conclusions: Treatment plans for patients aged 80 years old and above should be tailored to the patient’s colorectal cancer presentation, comorbidity status and life expectancy, weighing the impact of cancer treatments on the patient’s short- and long-term outcomes.

## Simple Summary

Colorectal cancer is the most common gastrointestinal neoplasm and affects older patients aged over 80 years old in about 10% of cases. The treatment of these patients is more complicated than the treatment of younger patients due to their comorbidities and frailty. This research examines a cohort of older colorectal cancer patients to identify characteristics and treatment strategies used in their management and compares them with those in younger colorectal cancer patients.

## 1. Introduction

Colorectal cancer is the most prevalent gastrointestinal malignancy and affects about 2 million people worldwide [1]. It ranks third in incidence after lung and breast cancers and second in mortality after lung cancer globally. Therefore, colorectal cancer remains a prominent public health concern, despite progress in developing treatments that have improved survival outcomes for patients with metastatic disease [2]. Despite the introduction and availability of novel targeted therapies and immunotherapy for a sub-set of colorectal cancer patients, most patients rely still on cytotoxic chemotherapy, both in early and metastatic settings. Although colorectal cancer diagnosis in younger persons has been increasing in recent years, the incidence in older populations is the highest, increasing with age [3,4,5]. The incidence of colorectal cancer per 100,000 population increases from 66.1 in those between 55 and 60 years old, to 214.7 for those 80 to 85 years old, and 234.7 for people above 85 years old [5]. In addition, the older population has frequent comorbidities and frailty that make oncologic treatments for these cancers more toxic and difficult to administer [6]. Given their frailty, older patients are often considered poor surgical candidates and many clinicians are also reluctant to treat this population of older adults with cytotoxic chemotherapies [6].

Early localized colorectal cancer may be cured with surgical resection [7]. More advanced localized cancers with deep penetration into the colonic wall or with extension to regional mesenteric lymph nodes carry a higher risk of recurrence after surgical resection, reaching 50% to 60% for lymph node-positive cancers, and require adjuvant chemotherapy to decrease this risk and improve the chance of a cure [2]. As a result, many patients with localized colorectal cancers have an oncologic indication for adjuvant fluoropyrimidine-based chemotherapy. Older patients tend to undergo screening for colorectal cancer less often, and are frequently diagnosed with symptomatic disease, which is more often of a more advanced stage [8]. Therefore, a combination of more advanced disease and an inability to receive, or reluctance to administer, adjuvant treatment in older colorectal cancer patients may lead to inferior outcomes. On the other hand, toxic treatments may carry an unacceptably high risk of adverse effects in older patients, especially those with significantly reduced organ reserves, and in these cases toxic treatments with the potential for serious harm should be avoided, even when a high recurrence risk is present [9]. Unfortunately, in such cases, elderly patients are left only with palliative options, and suffer short survivals and decreased quality of life due to progressive cancer symptoms.

In this report, we present the characteristics, treatments and outcomes of patients older than 80 years old diagnosed with localized colorectal cancer in our center, and compare them with patients of younger age presenting with the same disease, with the aim of better understanding the differences that characterize the disease in younger and older patients and optimizing outcomes.

## 2. Patients and Methods

Patients’ electronic records were retrospectively reviewed, and patients over 80 years old who had been treated for colorectal cancer in our cancer center between 2016 and 2023 were identified for inclusion in the study. The length of the reviewed period of medical records for the study was based on the prevalence of octogenarians in populations of colorectal cancer patients in the literature, with the aim of obtaining a sample size of more than 100 patients. This number of patients (and a similar number of controls) was considered sufficient for revealing major clinically relevant differences between the groups. The records of included patients were assessed, and demographic, cancer and outcome characteristics were extracted and documented in the study database. Data for a control cohort of younger colorectal cancer patients aged 65 to 75 treated during the same time period, matched by date of diagnosis, were extracted and reviewed. Information of interest that was recorded in the study database included the location, grade and stage of tumors, laboratory parameters, tumor markers, such as Carcinoembryonic Antigen (CEA), initial patient presentation and comorbidities. Comorbidities were assessed through reviewing medical notes, laboratory studies and the pharmacologic therapies that patients were receiving. Data on patient treatments and outcomes, including surgical treatment, neoadjuvant and adjuvant chemotherapy, dose of chemotherapy and adverse effects, and radiation therapy, were also extracted from electronic medical records. Adverse effects from treatments were evaluated from medical records, and, in cases in which the adverse effect was not directly attributed to treatment, the timing of occurrence and plausibility of relatedness were considered in attributing the side effects to the treatment. Overall survival was calculated as the time from diagnosis to the patient’s death, or censored to the time of the last follow-up. Progression-free survival was calculated as the time from diagnosis to the time of disease recurrence, or was censored to the time of the last follow-up without recurrence.

The primary data recorded in the study database were used to calculate descriptive statistics, including means and medians, standard deviation and range. Differences between the older and younger colorectal cancer group in the clinical and biologic parameters of interest were statistically compared with the χ^2^ test or Fisher’s exact test. Kaplan–Meier survival curves were constructed for the visual evaluation of the overall survival (OS) and progression-free survival (PFS) of the two groups of patients. The Log Rank test was employed for the statistical comparison of the PFS and OS curves. All the resulting *p* values were considered to be significant at the level of *p* < 0.05. Statistical calculations were performed using GraphPad (GraphPad Software, Boston, MA, U.S.A., www.graphpad.com/quickcalcs/) (URL last accessed on 10 November 2024). The Kaplan–Meier curves and Log Rank test were performed using an online tool based on R (statskingdom.com/Kaplan–Meier.html) (URL last accessed on 11 November 2024). The protocol of the research was approved by the Research Ethics Board of the hospital (Research Ethics Board number 2021-05-01).

## 3. Results

One hundred and four patients aged 80 years old and older (older group) were diagnosed with non-metastatic colorectal cancer and treated in a single cancer center between 2016 and 2023. A similar group of one hundred and six patients aged 65–75 (younger group) were diagnosed and treated during the same time period, and were included as controls. The mean age of the older group was 85 years old (range: 80–95). The mean age of the younger group was 71 years old (range: 65–75) (Table 1). There was no significant difference between male versus female sex in each group. Patients in the younger group more frequently had a family history of various cancers other than colorectal cancer (65.1%) when compared to the older group (53.1%), with this difference approaching statistical significance (*p* = 0.08, Table 1); however, no significant difference was observed regarding family history of colorectal cancer between the two groups. Colorectal cancer in the younger group was numerically more frequently diagnosed on screening compared to the older group (older group: 25.0%, younger group: 39.8%, *p* = 0.1). Conversely, initial presentation with symptoms of colorectal cancer was more frequent in the older group (bleeding or anemia: older group: 61.5%, younger group: 48.5%; bowel changes or obstruction: older group: 13.5%, younger group: 11.7%). Localized colorectal tumors also presented at similar anatomic locations between each group, with right-sided colon cancers being the most common site of tumor development (older group: 61.2%, younger group: 53.8%), followed by rectosigmoid and rectal tumors (older group: 25.2%, younger group: 27.3%) and then left-sided tumors (older group: 13.6%, younger group: 18.9%) (Table 1). No significant difference was observed between the older group and the younger group regarding the stage and grade of the cancer at initial diagnosis. In the older group, localized tumors were most commonly diagnosed as stage II malignancies (31.2%), whereas in the younger group, patients were more likely to be diagnosed with stage III malignancies (42.6%), although these differences were not statistically significant. Grade 3 tumors were the most common in both age groups (older group: 51.3%, younger group: 49.4%) (Table 1).

In examining the laboratory values at initial diagnosis between the older and younger group, no significant differences were observed in CEA levels, lactate dehydrogenase (LDH), albumin levels, platelet counts or random glucose levels between the older and younger study participants (Table 2). Elevated creatinine levels were found to be significantly more frequent in the older age group (15.4%) compared to the younger group (3.8%, *p* = 0.004) (Table 2).

Patients in the older age group were significantly less likely to receive neoadjuvant chemotherapy for rectosigmoid and rectal cancers (older group: 19.2%, younger group: 58.6%, *p* = 0.002), and adjuvant chemotherapy for stage II tumors (older group: 7.9%, younger group: 40.0%, *p* = 0.002), compared to their younger counterparts (Table 3). No significant difference was noted overall in adjuvant chemotherapy treatment for stage III tumors. In the older cohort who received adjuvant chemotherapy, a significant number of patients received reduced or modified doses of treatment (76.0%, *p* = 0.0001), compared to the younger cohort (10.0%). There was also a significant difference in the types of adjuvant chemotherapy used to treat stage II and III cancers between each age group (*p* = 0.00002). In the older cohort, monotherapy with Capecitabine was most commonly used (60.0%), followed by FOLFOX (5-FU/Folinic acid/Oxaliplatin) or CAPOX (Capecitabine/Oxaliplatin) (28.0%), and FOLFIRI (5-FU/Folinic acid/Irinotecan)/Bevacizumab) (12.0%). Combination therapy with FOLFOX or CAPOX was the most common treatment used in the younger cohort (82.0%), followed by Capecitabine (12.0%) and FOLFIRI/Bevacizumab (6.0%) (Table 3). Both neoadjuvant and adjuvant radiation therapy for rectosigmoid and rectal tumors were used significantly less in the older age group (older group: 34.6%, younger group: 65.5%, *p* = 0.02), whereas palliative radiation was significantly more common in the older cohort, although it was required in few cases in both groups (older group: 10.6%, younger group: 1.9%, *p* = 0.01). Older patients were also significantly less likely to undergo surgery for treatment of their colorectal cancer, compared to their younger counterparts (older group: 83.7%, younger group: 95.3%, *p* = 0.006) (Table 3).

In those patients who received chemotherapy, adverse effects were analyzed. Common adverse effects reported in both age groups included fatigue, neutropenia, peripheral neuropathy, hand–foot syndrome and cutaneous toxicities, bowel habit changes (constipation, diarrhea), nausea/vomiting, mucositis and others, including dehydration, visual changes, bleeding and dizziness (Table 4). Peripheral neuropathy was significantly more common in the younger age group (37.3%, *p* = 0.01), whereas other adverse effects (dehydration, bleeding) were reported significantly more often in the older age group (28.1%, *p* = 0.04) (Table 4).

Regarding comorbidities in each age group, some differences were also noticed. In the younger age group, the rate of obesity (BMI > 30) was significantly higher (older group: 28.8%, younger group: 46.2%, *p* = 0.01) (Table 5). In the older age group, hypertension, cardiovascular/neurovascular diseases and dyslipidemia were significantly more common. In contrast, the prevalence of diabetes was not different between patients of the two age groups (Table 5). Patients in the older group with cardiovascular/neurovascular comorbidities were less likely to have received combination chemotherapy (6.3% of cases) than patients in the older group without such comorbidities (17.1% of cases), although this difference did not reach statistical significance (*p* = 0.08).

**Figure 1:**
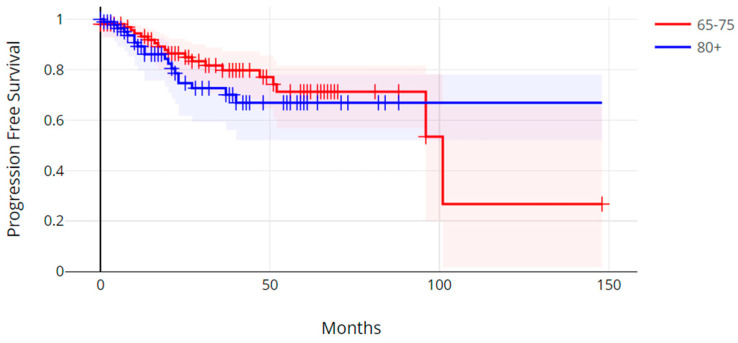
Progression-free survival of patients with colorectal cancer aged over 80 years old versus patients aged 65 to 75 years old. Log Rank test *p* = 0.3.

**Figure 2:**
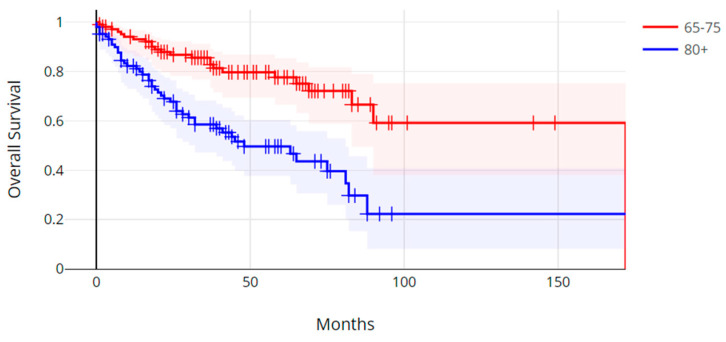
Overall survival of patients with colorectal cancer aged over 80 years old versus patients aged 65 to 75 years old. Log Rank test *p* = 0.00001.

The median progression-free survival in the older age group was 16.0 months (range, 0.3 months–8.9 months), and the median progression-free survival in the younger age group was 27.4 months (0.2 months–101.1 months). There was no statistically significant difference in progression-free survival between the two age groups (Log Rank *p* = 0.31) (Figure 1). The median overall survival (OS) in the older group was 26.3 months (range, 0.3 months–96.2 months), and the median overall survival in the younger age group was 39.7 months (range, 1.5 months–172.5 months) (Log Rank *p* = 0.00001) (Figure 2).

Table 6 shows the major differences in the presentation, comorbidities, treatments and adverse effects between the older and younger colorectal cancer patients, as observed in the cohorts of the study.

## 4. Discussion

Several of the most frequent cancers are more prevalent in older patients, and this trend is expected to increase with the aging of the population in western countries. About one in six adults are diagnosed with cancer at an age older than 80 years old [10]. Patients in this age group tend to receive treatments according to the standard of care for their specific type and stage of cancer less often, due to a perception of futility or excessive toxicity of treatments in the elderly. For example, a study from Canada showed that more than one-third fewer patients aged 80 or older were seen in surgical consultation for their cancer compared to cancer patients aged 65 years old and younger [10]. In addition, older patients were also less likely to be seen in medical and radiation oncology consultations. These trends suggest that older cancer patients may be denied valuable treatments that could potentially prolong their survival and improve the quality of their lives. On the other hand, it is well documented that all cancer treatments may more commonly have adverse outcomes in the elderly who have more comorbidities and reduced functional reserves. Surgical resection of localized cancers in octogenarians is associated with a perioperative mortality rate of 7% to 11% [11,12]. Complications related to surgery, such as operative leaks and infections, are not more common in older patients, but are more frequently fatal. Age- and disease-related sarcopenia and cachexia are prevalent in the elderly, and may lead to adverse outcomes with surgical complications. Cachexia, defined as weight loss of more than 5%, or of 2% to 5% when the body mass index is less than 20 kg/m^2^, was present in 36% of patients with localized colorectal cancer and a mean age of 79.6 years old [13]. The prevalence of sarcopenia was 13% in the same series. Chemotherapy is also more toxic for elderly patients, and in many cases it is omitted, even when there is an oncologic indication for its administration [14]. Tools to predict high-grade adverse effects in older patients have been proposed, and play a role in improving the anticipation of adverse outcomes in individual patients, who may be then counseled against treatment, or be treated with modified doses or enhanced surveillance and support [9]. Therefore, a strategy with the specific patient in view, and which considers not only their age, but also their general status and individual wishes for treatment, would better serve this more challenging-to-treat patient age group.

In our cohort of stage I to III colorectal cancer patients over 80 years old, there were no significant differences in the distribution of genders, location of the tumor in the colon or grade of the tumor compared to a younger cohort. A trend toward a lower incidence of family history of cancers and a lower prevalence of incidentally and screening-diagnosed cancers in the older group was present. This is related to the fact that, although screening colonoscopies decrease the incidence of colorectal cancer diagnosis, guidelines advise for selective-only screening over the age of 75 [15,16,17]. A decreased incidence of screening-diagnosed cancer has also been reported in other cancers, such as breast cancer, in the elderly, as guidelines advise against routine screening [18]. The two age groups did not differ in their baseline CEA, rate of thrombocytosis or hypoalbuminemia, but older patients more frequently had decreased renal function. Although younger patients had significant higher rates of obesity, and the rate of diabetes did not differ between the groups, older patients had a higher frequency of hypertension, cardiovascular disease and dyslipidemia. Significant differences were observed in treatments, with the older patients less frequently receiving all types of cancer therapies, besides palliative radiation. In addition, the type of adjuvant chemotherapy differed between the groups, with the majority of older patients receiving fluoropyrimidine-based monotherapy, and three-fourths of the patients having a reduced dose of chemotherapy. As this was a retrospective analysis, the treatment decisions had been made by the treating physicians in a non-randomized manner. Therefore, it cannot be ascertained by the current analysis whether the process of treatment allocation in individual patients was based on age considerations alone, or whether it considered other factors, such as comorbidities and frailty. However, the process of multi-parametric decision making is routine in oncology. Moreover, a significant proportion of older patients in the study (28%) had indeed received combination chemotherapy, suggesting that additional patient factors, in addition to age, were taken into consideration in deciding the most appropriate treatment for the individual patient. Despite the treatment differences, and despite that, as expected, older patients had worse overall survival than younger patients, progression-free survival did not differ significantly between the two groups. Given the similar progression-free survival in the two groups, and the fact that baseline characteristics were well balanced (Table 1 and Table 2), no multivariate analysis was performed.

The location of the cancer in the right or left colon is of biological and clinical importance, as right-sided cancers tend to more often be developing along the microsatellite instability pathway, and tend to have a better prognosis when localized. Some studies have reported a higher incidence of right-sided colon primaries in older patients, although this has not been observed by other investigators [14,19,20]. Given their better prognosis, stage II right-sided colon cancers with microsatellite instability may be managed without adjuvant treatment. Ongoing clinical studies are examining whether more advanced localized stages with microsatellite instability may be successfully treated with immunotherapy instead of chemotherapy, based on the success of these treatments in treating microsatellite-unstable metastatic disease [21]. In our series, a non-significant trend for a higher incidence of right-sided colon primaries in the older population was observed.

Although all cancer treatment modalities, including surgery, radiation and chemotherapy, were used less frequently in patients older than 80 years than in younger patients in our cohort, most patients received treatment appropriate for the stage of their cancer. For example, 83.7% of octogenarians and older patients had surgical resection of their colorectal cancer. The feasibility and acceptable outcomes of surgical resection of colorectal cancer in octogenarians and nonagenarians in contemporary practice have been documented, with post-operative 90-day survival rates of 98.1% and 98.1%, respectively, and post-operative 180-day survival rates of 93.1% and 88.9%, respectively [22].

Adjuvant chemotherapy is recommended for all stage III colorectal cancer patients that have an adequate performance status for receiving the treatment, and it was administered in 68.7% of octogenarians and older patients in our cohort. Monotherapy with capecitabine was favored in this population as a less toxic treatment, and dose adjustments were frequently performed. The overall rate of chemotherapy administration in our elderly cohort was higher than the rate reported in other series and databases which ranges from 3% to 25%, suggesting that, with appropriate dose and intensity adjustments, adjuvant therapy is feasible [14,23]. Adjuvant chemotherapy in patients over 80 years old with stage III colorectal cancer has been associated with improved survival. In a study on the United States National Cancer Database, patients over the age of 80 years old with stage III colorectal cancer who received adjuvant chemotherapy had a median overall survival of 61.7 months, while similar patients who did not receive adjuvant chemotherapy had a median overall survival of 35 months [24]. The progression-free survival outcomes observed in our older cohort suggest that less intense chemotherapy regimens may provide results that are similar to those obtained with combination adjuvant treatments employed in younger patients. Combination chemotherapy with a fluoropyrimidine and oxaliplatin is the standard of care in the adjuvant setting for stage III colorectal cancer, based on its superior efficacy compared to fluoropyrimidines alone [25]. However, the pivotal trials that established this standard excluded older patients over the age of 75 years old. Capecitabine monotherapy may be adequate as an adjuvant therapy for stage III colorectal cancer patients aged over 80 years old, and provides a survival benefit compared to no adjuvant treatment [26]. Geriatric tools to predict high-grade toxicity from chemotherapy in the elderly may provide further guidance for the advisability of adjuvant treatment in the elderly, and could serve as a safeguard against advising therapy in patients predicted to have an unacceptably high risk of toxicity [27]. A tool devised by the Cancer and Aging Research Group (CARG) provides a validated scale for the prediction of chemotherapy toxicity in the elderly, and is endorsed by the American Society of Clinical Oncology (ASCO) [28]. The CARG tool assigns a low, intermediate or high risk for toxicity to geriatric patients aged above 70 years old, based on 11 parameters, which include an age above 72 years old, a diagnosis a gastrointestinal or genitourinary cancer, the dose and number of chemotherapy drugs, anemia, renal insufficiency, hearing loss, previous falls, an inability to take medications, mobility restrictions and social activity [28]. A simpler prediction tool based on just four parameters—Eastern Co-operative Oncology Group (ECOG) performance status, hypoalbuminemia, renal insufficiency and presence of a metastatic cancer—has been proposed and found to perform similarly to the CARG tool [29,30]. However, this tool has not been validated more broadly. The European Society of Medical Oncology, in collaboration with the International Society of Geriatric Oncology (SIOG), has advocated, in a position paper, for the use of geriatric assessments for the optimization of the management of geriatric cancer patients [31].

The current study has certain limitations. The cohort was studied retrospectively and was recruited from a single cancer center. Although the current study included over one hundred stage I to III colorectal cancer patients older than 80 years old, which is one of the highest numbers of patients studied in this population reported in the literature, the number of included patients was still comparatively small. The source records had incomplete data on patients’ performance status and quality of life evaluations. Therefore, an analysis of these parameters could not be performed in the current study. Moreover, the role of comorbidities in the difference in OS between the two groups of patients was not assessed. No molecular data regarding mismatch repair status and other mutations were available in the current report. However, other studies have confirmed a higher prevalence of mismatch repair deficiency in colorectal cancer patients and a higher prevalence of cases with a high tumor mutation burden in this population [6,32,33]. Both molecular markers provide opportunities for treatment with checkpoint inhibitor immunotherapy in the metastatic setting, and this treatment could be expanded in the localized setting if ongoing trials confirm its benefit. Common cancer-associated alterations of colorectal cancer, including mutations in the tumor suppressors *APC*, *TP53* and in the oncogenes *KRAS* and *PIK3CA*, have shown no significant differences in prevalence between age groups [32,33,34].

In conclusion, the treatment of stage I to III colorectal cancer in octogenarians is a conundrum frequently faced by physicians treating cancer in daily practice. Older colorectal cancer patients differ from younger patients in their organ function reserves, notably their renal function, which limits their ability to receive more intensive treatments. Indeed, we have observed lower rates of chemotherapy use and a lower intensity of treatments in the older patients. Particularly, the presence of comorbidities affects the intensity of the chemotherapy used. A multidisciplinary approach should be influenced by the individual patient’s general status and wishes, and should take advantage of all available therapies tailored to the specific patient. The optimization of treatment has the potential to provide beneficial outcomes of localized colorectal cancer in octogenarians and older patients that are similar to those obtained in younger patients.

## Figures and Tables

**Table 1:** Patient and disease characteristics in colorectal cancer patients aged over 80 years old versus patients aged 65–75 years old.

Characteristic	All (%) (*n* = 210)	Group > 80 Years Old (%) (*n* = 104)	Group 65–75 Years Old (%) (*n* = 106)	*p*
**Mean Age (Range)**		85 (80–95)	71 (65–75)	
**Gender**				
Male	116 (55.2%)	53 (51.0%)	63 (59.4%)	0.21
Female	94 (44.8%)	51 (49.0%)	43 (40.6%)	
**Family History of Colon Cancer**				
Yes	44 (21.6%)	22 (22.4%)	22 (20.8%)	0.77
No	160 (78.4%)	76 (77.6%)	84 (79.2%)	
N/A	6	6	0	
**Family History of Other Cancers**				
Yes	121 (59.3%)	52 (53.1%)	69 (65.1%)	0.08
No	83 (40.7%)	46 (46.9%)	37 (34.9%)	
N/A	6	6	0	
**Presentation of Disease**				
Incidental/Screening	67 (32.4%)	26 (25.0%)	41 (39.8%)	0.10
Anemia/Bleeding	114 (55.1%)	64 (61.5%)	50 (48.5%)	
Bowel Changes/Obstruction	26 (12.5%)	14 (13.5%)	12 (11.7%)	
N/A	3	0	3	
**Location of Tumor**				
Right	120 (57.4%)	63 (61.2%)	57 (53.8%)	0.48
Left	34 (16.3%)	14 (13.6%)	20 (18.9%)	
Rectal/Rectosigmoid	55 (26.3%)	26 (25.2%)	29 (27.3%)	
N/A	1	1	0	
**Stage at Diagnosis**				
I	40 (21.3%)	17 (19.5%)	23 (22.7%)	0.45
II	73 (38.8%)	38 (43.7%)	35 (34.7%)	
III	75 (39.9%)	32 (36.8%)	43 (42.6%)	
N/A	22	17	5	
**Grade of Tumor**				
1	24 (14.2%)	14 (17.5%)	10 (11.2%)	0.37
2	60 (35.5%)	25 (31.2%)	35 (39.4%)	
3	85 (50.3%)	41 (51.3%)	44 (49.4%)	
N/A	41	24	17	

**Table 2:** Laboratory values at initial presentation in patients with localized colorectal cancer aged over 80 years old versus patients aged 65 to 75 years old.

	All (%) (*n* = 210)	Group > 80 Years Old (*n* = 104)	Group 65–75 Years Old (*n* = 106)	*p*
**CEA**				
≤4.7 µg/L	129 (70.5%)	66 (72.5%)	63 (68.5%)	0.55
>4.7 µg/L	54 (29.5%)	25 (27.5%)	29 (31.5%)	
N/A	27	13	14	
**LDH**				
≤210 U/L	181 (90.0%)	90 (90.9%)	91 (89.2%)	0.69
>210 U/L	20 (10.0%)	9 (9.1%)	11 (10.8%)	
N/A	9	5	4	
**Albumin**				
<35 g/L	59 (29.2%)	33 (33.0%)	26 (25.5%)	0.24
≥35 g/L	143 (70.8%)	67 (67.0%)	76 (74.5%)	
N/A	8	4	4	
**Platelets**				
≤400 × 10^9^/L	185 (88.9%)	93 (90.3%)	92 (87.6%)	0.54
>400 × 10^9^/L	23 (11.1%)	10 (9.7%)	13 (12.4%)	
N/A	2	1	1	
**Glucose**				
≤7 mmol/L	87 (52.4%)	45 (97.6%)	42 (50.0%)	0.53
>7 mmol/L	79 (47.6%)	37 (2.4%)	42 (50.0%)	
N/A	44	22	22	
**Creatinine**				
≤130 µmol/L	189 (90.4%)	88 (84.6%)	101 (96.2%)	0.004
>130 µmol/L	20 (9.6%)	16 (15.4%)	4 (3.8%)	
N/A	1	0	1	

**Table 3:** Cancer treatments in patients with colorectal cancer aged over 80 years old versus patients aged 65 to 75 years old.

Treatment	All (%)	Group > 80 Years Old (%)	Group 65–75 Years Old (%)	*p*
**Neoadjuvant chemotherapy (rectosigmoid/rectal)**				
Yes	22 (40.0%)	5 (19.2%)	17 (58.6%)	0.002
No	33 (60.0%)	21 (80.7%)	12 (41.4%)	
**Adjuvant chemotherapy (stage II)**				
Yes	17 (23.3%)	3 (7.9%)	14 (40.0%)	0.002
No	56 (76.7%)	35 (92.1%)	21 (60.0%)	
**Adjuvant chemotherapy (stage III)**				
Yes	58 (77.3%)	22 (68.7%)	36 (83.7%)	0.13
No	17 (22.7%)	10 (31.3%)	7 (16.3%)	
**Type of adjuvant chemotherapy (stage II and III)**				
Capecitabine	21 (28.0%)	15 (60.0%)	6 (12.0%)	0.00002
FOLFOX or CAPOX	48 (64.0%)	7 (28.0%)	41 (82.0%)	
FOLFIRI/Bevacizumab	6 (8.0%)	3 (12.0%)	3 (6.0%)	
**Adjuvant chemotherapy dose reduction**				
Yes	24 (32.0%)	19 (76.0%)	5 (10.0%)	0.0001
No	51 (68.0%)	6 (24.0%)	45 (90.0%)	
**Neoadjuvant/adjuvant radiation (rectosigmoid/rectal)**				
Yes	28 (50.9%)	9 (34.6%)	19 (65.5%)	0.02
No	27 (49.1%)	17 (65.4%)	10 (34.5%)	
**Palliative radiation**				
Yes	13 (6.2%)	11 (10.6%)	2 (1.9%)	0.01
No	197 (93.8%)	93 (89.4%)	104 (98.1%)	
**Surgery**				
Yes	188 (89.5%)	87 (83.7%)	101 (95.3%)	0.006
No	22 (10.5%)	17 (16.3%)	5 (4.7%)	

**Table 4:** Chemotherapy-associated adverse effects in patients with colorectal cancer aged over 80 years old versus patients aged 65–75 years old.

Adverse Effect	All (%) (*n* = 91)	Group > 80 Years Old (%) (*n* = 32)	Group 65–75 Years Old (%) (*n* = 59)	*p*
**Fatigue**				
Yes	19 (20.9%)	7 (21.9%)	12 (20.3%)	1.00
No	72 (79.1%)	25 (78.1%)	47 (79.7%)	
**Neutropenia (grade 4)**				
Yes	4 (4.4%)	0 (0.00%)	4 (6.8%)	0.3
No	87 (95.6%)	32 (100.00%)	55 (93.2%)	
**Peripheral neuropathy**				
Yes	25 (27.5%)	3 (9.4%)	22 (37.3%)	0.01
No	66 (72.5%)	29 (90.6%)	37 (62.7%)	
**Hand–foot syndrome/skin changes**				
Yes	19 (20.9%)	10 (31.3%)	9 (15.2%)	0.10
No	72 (79.1%)	22 (68.7%)	50 (84.8%)	
**Bowel changes (diarrhea, constipation)**				
Yes	34 (37.4%)	12 (37.5%)	22 (37.3%)	1.00
No	57 (62.6%)	20 (62.5%)	37 (62.7%)	
**Nausea/vomiting**				
Yes	18 (19.8%)	5 (15.6%)	13 (22.0%)	0.59
No	73 (80.2%)	27 (84.4%)	46 (78.0%)	
**Mucositis**				
Yes	7 (7.7%)	3 (9.4%)	4 (6.8%)	0.69
No	84 (92.3%)	29 (90.6%)	55 (93.2%)	
**Other (dehydration, vision changes, bleeding, dizziness)**				
Yes	15 (16.5%)	9 (28.1%)	6 (10.2%)	0.04
No	76 (83.5%)	23 (71.9%)	53 (89.8%)	

**Table 5:** Comorbidities in patients with colorectal cancer aged over 80 years old versus patients aged 65–75 years old.

Comorbidities	All (%) (*n* = 210)	Group > 80 Years Old (%) (*n* = 104)	Group 65–75 Years Old (%) (*n* = 106)	*p*
**Obesity (*n* = 210)**				
BMI > 30	79 (37.6%)	30 (28.8%)	49 (46.2%)	0.01
BMI ≤ 30	131 (62.4%)	74 (71.2%)	57 (53.8)	
**Diabetes (*n* = 209)**				
Yes	67 (31.9%)	36 (34.6%)	31 (29.2%)	0.43
No	142 (67.6%)	68 (65.4%)	74 (69.8%)	
**Hypertension (*n* = 209)**				
Yes	158 (75.2%)	87 (83.7%)	71 (66.9%)	0.007
No	51 (24.8%)	17 (16.3%)	34 (32.1%)	
**Cardiovascular/neurovascular disease (*n* = 209)**				
Yes	99 (47.1%)	68 (65.4%)	31 (29.2%)	0.0001
No	110 (52.4%)	36 (34.6%)	74 (69.8%)	
**Dyslipidemia (*n* = 209)**				
Yes	108 (51.4%)	62 (59.6%)	46 (43.4%)	0.02
No	101 (48.6%)	42 (40.4%)	59 (55.7%)	

**Table 6:** The main differences between the older (aged over 80 years old) and younger (aged 65 to 75 years old) colorectal cancer patients observed in this study.

Characteristic	Comparison	*p* Value
Demographics		
Family history of other cancers besides colorectal cancer	Trend of higher prevalence in younger group	0.08
Presentation of disease	Trend of more frequent presentation with anemia or bleeding in older group	0.1
Laboratory evaluation		
Creatinine	Older group more frequently (15.4% of cases) had elevated values than younger group (3.8% of patients)	0.004
Treatments		
Neoadjuvant chemotherapy in patients with rectal/rectosigmoid cancer	More commonly administered in younger group (58.6% of patients) than in older patients (19.2%)	0.002
Adjuvant chemotherapy	More frequent use of adjuvant chemotherapy for stage II cancers in younger group (40% versus 7.9% of older patients), but no difference in use of adjuvant chemotherapy for stage III patients	0.002
Type of adjuvant chemotherapy	More frequent use of adjuvant capecitabine monotherapy in older group (60% versus 12% in younger group)	0.00002
Adjuvant chemotherapy dose reduction	More frequently performed in older group (76% versus 10% in younger group)	0.0001
Neoadjuvant/adjuvant radiation in patients with rectal/rectosigmoid cancer	More frequently given in younger patients (65.5% of patients versus 34.6% of patients in older group)	0.02
Surgery	Fewer patients in older age group were treated surgically (83.7% versus 95.3% in younger group)	0.006
Adverse effects		
Peripheral neuropathy	More frequent in younger patients (37.3%) than in older patients (9.4%), consistent with more frequent use of oxaliplatin in this group	0.01
Hand–foot syndrome	Trend for higher incidence in older group (31.3% versus 15.2% of patients in younger group)	0.10
Comorbidities		
Obesity	Higher prevalence in younger group (46.2%) than in older patients (28.8%)	0.01
Hypertension, cardiovascular disease and dyslipidemia	More prevalent in younger patients	0.007, 0.0001, 0.02

## Data Availability

All the data generated in this study are presented in the article, and no additional data are available.
